# Clinical Course and Outcomes of Late Kidney Allograft Dysfunction

**DOI:** 10.1155/2016/7401808

**Published:** 2016-07-10

**Authors:** Viktor Denisov, Vadym Zakharov, Anna Ksenofontova, Eugene Onishchenko, Tatyana Golubova, Sergey Kichatyi, Olga Zakharova

**Affiliations:** Transplant Center, Regional Hospital, Illyicha Avenue 14, Donetsk 83000, Ukraine

## Abstract

*Background.* This study is provided to increase the efficiency of the treatment of kidney transplant recipients by predicting the development of the late allotransplant dysfunction.* Methods*. 330 patients who have lived for more than one year with functioning kidney allograft were evaluated. To predict the subsequent duration of the well-functioning of allotransplant the prognostic significance of 15 baseline clinical and sociodemographic characteristics on the results of the survey one year after transplantation was investigated. The result was considered to be positive in constructing the regression prognostication model if recipient lived more than 3 years from the time of transplantation.* Results*. It was established that more late start of renal allograft dysfunction after transplantation correlates with the more time it takes till complete loss of allograft function. Creatinine and hemoglobin blood concentration and the level of proteinuria one year after transplantation within created mathematical model allow predicting the loss of kidney transplant function three years after the transplantation. Patients with kidney transplant dysfunction are advised to renew the program hemodialysis upon reaching plasma creatinine concentration 0.5–0.7 mmol/L.* Conclusion*. Values of creatinine, hemoglobin, and proteinuria one year after transplantation can be used for subsequent prognostication of kidney transplant function.

## 1. Introduction

Significant improvement of kidney transplantation results in the early postoperative period during recent decades is accompanied by the expansion of the pool of patients with long periods of observation. The most difficult problem of the late postoperative period is chronic nephropathy of the transplant [1–6]. Late dysfunction of kidney transplant is polygenic and multistep process that leads to returning to dialysis or patient's death [2, 7, 8]. The steadily increasing number of kidney transplants in the future is one of the significant sources of expanding of the pool of dialysis patients due to a new and rather specific form of chronic renal failure, a chronic allograft nephropathy. Effective treatment must not only predict the rate of growth of transplanted kidney dysfunction but also have guidelines for immunosuppression and determine the optimum timing of the transfer of transplant recipients on dialysis treatment program [9–21].

The objective of this study is to increase the efficiency of the treatment of recipients in the late period after kidney transplantation by estimating the probability of loss of function of kidney transplant in the long-term period after transplantation.

## 2. Patients and Methods

330 patients who had been receiving renal replacement therapy since 1987 till 2012 and living longer than one year with a functioning kidney transplant were selected in the study group. There were 61.2% males and 38.8% females. Age was ranging from 6 to 71 years. The median recipient age was 42.6 years. The main disease was glomerulonephritis (78.2%). Polycystic disease, diabetes, or other causes were more seldom. Most of them were on hemodialysis (96%); 13 patients received preemptive transplant. 56% of transplants were performed from deceased donors and 44% from the living donors. The “Custodiol HTK Solution” was used for cold storage of kidney transplants. The cold ischemia time was no more than 24 hours. Immunosuppression (at the initial stage of our work) consisted of cyclosporine, azathioprine, and steroids. Later (since the year 2000) in all cases of induction anti-CD-25 monoclonal or other depleting antibodies were used. Immunosuppression maintenance spectrum was added by tacrolimus, prolonged-release tacrolimus, mycophenolate mofetil, mycophenolate sodium, or everolimus.

The duration of observation after transplantation averaged 6.5 ± 1.4 years. Clinical and laboratory monitoring of the patient and kidney transplant were usually carried out on outpatient basis. Surveys were carried out depending on the period after kidney transplantation with a frequency of once a month up to 2–4 times per year. Particular attention was paid to the level of blood pressure, hemoglobin in the full blood count, creatinine concentration in blood plasma, and daily protein excretion. If necessary, sonography of transplant was performed.

We considered as appropriate the immunosuppression in which concentration of immunosuppressants in the blood was within the “therapeutic range” and the number of leukocytes in full blood analysis corresponded to normal.

The diagnosis of anemia was made at hemoglobin concentration of less than 130 g/L for men and less than 120 g/L for women. The blood pressure less than 140/90 mm Hg was considered as normal, blood pressure more than 140–159/90–99 mm Hg was considered as moderately elevated, and the blood pressure more than 160/100 mm Hg was considered as high. The degree of proteinuria was assessed by daily urinary protein excretion: minimum: loss of protein <0.5 g/day, moderate: 0.5–1 g/day, and severe: more than 1.0 g/day. In order to standardize the results, as a starting point of late dysfunction we conventionally adopted the increase of creatinine level to 0.3 mmol/L in plasma for the first time detected after kidney transplantation. We used SF-36 scale to assess patients' quality of life.

In order to identify the features that can be used to assess the degree of risk of death with a functioning transplant or return to the program hemodialysis the method of constructing of mathematical predicting models has been used.

To predict the subsequent duration of the well-functioning of allotransplant the prognostic significance of 15 baseline clinical and sociodemographic characteristics on the results of the survey one year after transplantation was investigated.

We studied age of the recipient at the time of renal transplantation, primary diagnosis, which led to the development of end-stage renal failure, type of donation (cadaveric or living), duration of hemodialysis treatment before kidney transplantation (in years), the presence of an induction immunosuppressive therapy with antibodies, the presence of hepatitis B or C infection at time of transplantation, education (primary school, general secondary education, or university), type of residence (urban, rural), number of previous transplantations (0, 1, 2, or 3), the concentration of creatinine in the blood one year after transplantation (mmol/L), blood pressure one year after transplantation (mm Hg), the concentration of hemoglobin in the blood one year after transplantation (g/L), proteinuria one year after transplantation (none, minimal: <0.5 g/L, moderate: 0.5–1 g/L, expressed: >1 g/L), and the duration of satisfactory function of renal transplant (in years) to death with a functioning graft or to return to the program hemodialysis.

The choice of factors taken as a basis was determined, first of all, by their accessibility to the wide range of physicians having the recipients in the late postoperative period under supervision. That is why we studied the possibility to use the most informative ones to predict the loss of kidney transplant function.

To assess the data obtained we used methods of parametric statistics including definition of their accuracy according to Student's criterion.

To reveal the most informative signs at assessing the degree of death risk with the functioning transplant or returning to programmed hemodialysis, for the purpose of possible correction of emergent threat, Cox multiple-factor regression model with the calculation of correlation coefficients was used. In this model the influence of each factor under study is characterized by an assessed coefficient which reflects the specific weight of the factor itself in the complex of assumed influence. The connection between studied separate risk factors of kidney transplant function loss was analyzed with the use of conjugation tables and by means of calculating the correlation coefficients.

In constructing of regression model of prognostication the result was considered positive if the recipient lived for more than 3 years from the time of transplantation.

Analysis of *β*-coefficients of the generalized regression model for the forecast was performed. On a dedicated set of features predictive model was created and optimized after model's decision-rejection threshold training.

Statistical packet SPSS was used for data processing.

## 3. Results

Standardization of all stages of pre-, intra-, and postoperative period facilitated an increase in one-year survival rate of kidney transplants especially in separate years of the last decade till 100%. In the late period in a year or later after the kidney transplant recipient's quality of life was close to the quality of life of healthy people and is significantly higher than the patients who continued dialysis (Table 1).

So the quality of life after transplantation was largely determined by the presence or absence of renal transplant dysfunction which dominated in the structure of adverse outcomes in transplant transplantations in late postoperative period. This is the reason why all the necessary measures should be carried out including prognostication and influence upon the modifying factors to ensure the stable kidney transplant function. Development of renal allograft dysfunction usually associated with anemia, hypertension, proteinuria, infections, and other adverse events.

**Figure 1:**
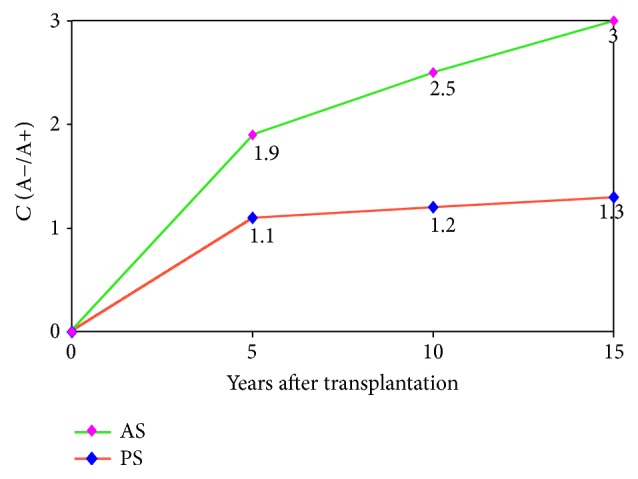
Correlation (*C*) of long-term survivability of patients (PS) and allografts (AS) depending on absence or presence of anemia (A−/A+).

In the analysis of the anemia prevalence in the late period after kidney transplantation and its correlation with survival of patients and transplants it is revealed that before kidney transplantation 98.7% of patients had signs of anemia. One year after kidney transplantation 20% of patients had anemia signs, three years after 28%, in 5 years 37%, in 10 years 45%, and in 15 years 46%. Functional survival of patient's transplants with normal levels of hemoglobin in the blood after 15 years of the operation was three times higher than that in the group of patients with anemia (Figure 1).

**Figure 2:**
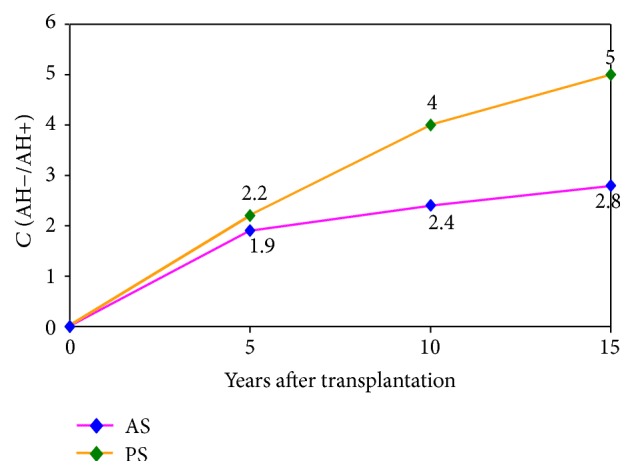
Correlation (*C*) of long-term survivability of patients (PS) and allografts (AS) depending on absence or presence of arterial hypertension (AH−/AH+).

Before kidney transplantation 1.8% of patients had normal blood pressure (BP), one year after kidney transplantation normal blood pressure occurred in 27% of patients, after 3 years, in 31% of patients, after 5 years, in 37% of patients, after 10 years, in 45% of patients, and after 15 years, in 54% of patients. Functional survival in patient's transplants with normal blood pressure after 15 years was 2.8 times higher than that in the group of patients with hypertension, and patients survival rate was almost 5 times higher (Figure 2). Our data also confirm the close connection between the severity of proteinuria, the presence of symptoms of chronic nephropathy of transplant, and the rate of its progression.

The average duration of transplant functioning from the moment of the detection of minimal proteinuria was 4.3 ± 2.5 years, with moderate proteinuria it was 2.7 ± 2.2 years, and in severe proteinuria it was 0.8 ± 0.3 years.

We found out that the rate of chronic nephropathy development in renal transplant depended on the duration of satisfactory function of renal transplant.

Patients who had signs of late dysfunction one year after transplantation returned to hemodialysis in 19.4 ± 2.6 months after the first recorded increase of the plasma creatinine up to 0.3 mmol/L.

Patients who had symptoms of late dysfunction 2-3 years after transplantation returned to hemodialysis in 40.0 ± 0.6 months after the first recorded increase of the plasma creatinine up to 0.3 mmol/L.

Patients who had symptoms of late dysfunction 5 years after transplantation returned to hemodialysis in 44.5 ± 0.9 months after the first recorded increase of the plasma creatinine up to 0.3 mmol/L.

In order to predict the duration of satisfactory function of the transplanted kidney we investigated the predictive value of 15 basic clinical laboratory and sociodemographic variables of the survey one year after transplantation (Table 2).

233 (70.6%) kidney transplant patients with functioning graft have been living over three years. Analysis of *β*-coefficients of the generalized regression model showed that three characteristics can be deemed the most significant (*P* < 0.001) for the forecast: blood creatinine and hemoglobin level and degree of proteinuria.

It was found that three signs can be the most significant (*P* < 0.001): creatinine concentration in the blood (*X*_1_), hemoglobin concentration in the blood (*X*_2_), and severity of proteinuria (*X*_3_).

The resulting predictive model using these factors was described by the equation: *Y* = − 0.967 × *X*_1_ + 0.00955 × *X*_2_ − 0.143 × *X*_3_ − 0.068, where *X*_1_ is creatinine (mmol/L), *X*_2_ is hemoglobin (g/L), and *X*_3_ is proteinuria in a year after transplantation (g/L). *Y* is a prognostic criterion. The value *Y*_crit_ = 0.435 is obtained. If as a result of calculation within the created model *Y* value is less than *Y*_crit_, a negative result is predicted (a loss of transplant function in three years from the time of transplantation) and otherwise a positive (favorable) result is predicted.

## 4. Discussion

**Figure 3:**
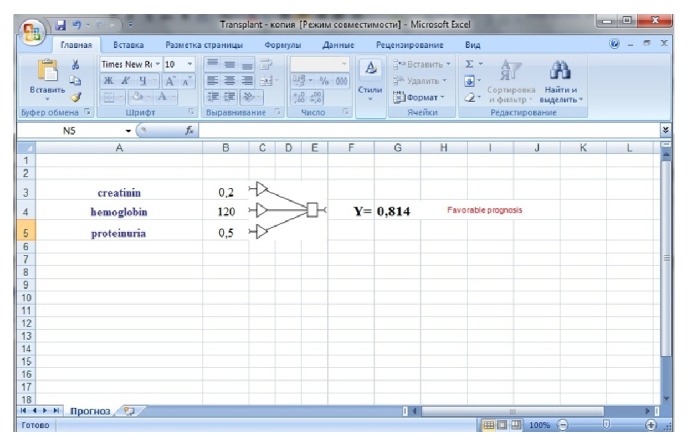
The interface of mathematical model for prognostication of kidney transplant function during the period of three years after the transplantation according to the results of investigation one year after the operation.

Determination of creatinine and hemoglobin level in the blood as well as the concentration of protein in the urine in one year after kidney transplantation with the calculation of prognostic criterion predicts the loss of renal allotransplant function in 3 years after surgery. The advantages of the method are the possibility of quantitative forecasting of renal allotransplant losses which is based not only on its excretory function assessment, but also on an assessment of other characteristics that may have important prognostic value and does not always directly correlate with changes in its excretory function. In order to assess the risk of death with a transplant functioning or return to the program hemodialysis the predictive model was implemented in tabular processor Excel. For the use of the model it is quite enough to input the value of the given indices. Calculation and prognosis will be automatically done in the electronic table (Figure 3).

The calculator designed by us has been patented (http://uapatents.com/4-68339-sposib-prognozuvannya-vtrati-funkci-nirkovogo-transplantata.html) and is available on the Internet (https://yadi.sk/i/w9DaT4YrsFRnZ). The accuracy of prediction of renal transplant function loss three years after transplantation was 92%.

Progression of chronic renal dysfunction of the transplant is accompanied by the simultaneous loss of the benefits of a successful transplantation and the growth of problems due to immunosuppression. Based on a retrospective analysis of results of treatment of kidney transplant of the recipients with blood creatinine higher than 0.3 mmol/L, we adhere to the following principles in the correction of immunosuppression which allow decreasing the rate of chronic dysfunction of the transplant development or decreasing the risk of complications in case of loss of its function.

1. Do not prescribe high doses of steroids and do not have the steroid pulse therapy.
2. Do not increase the dose of received cyclosporine or tacrolimus and stop medication if there is an increase in nephropathy.
3. Continue immunosuppression with medicines of mycophenolic acid which are not nephrotoxic.
4. Enhance monitoring of immunosuppression and prevention of infectious complications.
5. Cancel immunosuppression at returning to hemodialysis treatment. Cancellation of steroids should be done gradually, sometimes for several months. When the discomfort is associated with transplant (temperature, pain in the projection of the transplanted kidney, and hematuria) short courses of low doses of steroids administered orally or intravenously can be effective.

According to plasma concentration of creatinine at the return to hemodialysis the patients were divided into 3 groups. In the first group the creatinine concentration in blood plasma was 0.5–0.69 mmol/L, in the 2nd group concentration in blood plasma was 0.7–1.0 mmol/L, and in the third group concentration in blood plasma was more than 1.0 mmol/L.

Dates of the return of transplant recipients with delayed renal transplant dysfunction are largely dependent on the psychological state of the patient, severity of depression, the desire to ensure the irreversibility of the transplanted kidney dysfunction, and fear that the dialysis will contribute to the deterioration of renal transplant function.

The survival rate of patients of the first group after return to hemodialysis was 7.4 + 2.8 years, and in the second and third groups it was, respectively, 5.3 ± 3.2 and 2.8 + 2.6 years.

In general, the noninvasive prediction of loss of renal transplant function based on quantitative criteria which diversely characterized the state of renal transplant enabled timely influence on modifiable risk factors for dysfunction of the transplanted kidney, correct immunosuppression, or return kidney transplant recipients to dialysis treatment.

## 5. Conclusions

1. Our experience of renal transplantation confirms the principle possibility to achieve a guaranteed high level of patient's rehabilitation. The quality of medical and social rehabilitation after kidney transplantation is comparable with healthy individuals and is much better than the patients being treated with hemodialysis.
2. The results of kidney transplantation are largely determined by the presence of renal allotransplant dysfunction which dominated in the structure of the causes of an unfavorable outcome of kidney transplantation in the late postoperative period. More later development of the kidney transplant dysfunction correlated with the greater duration of the period prior to the loss of its function.
3. The absence of anemia and proteinuria after kidney transplantation allows counting on a significant improvement in survival of renal allotransplant and a recipient. Correction of blood pressure also allows counting on a significant improvement in the results of renal transplantation in the late postoperative period.
4. Determination of creatinine and the hemoglobin concentration in blood and the protein concentration in urine one year after transplantation with the calculation of prognostic index allows predicting the loss of renal allotransplant function in 3 years after transplantation with accuracy up to 92%. The constructed mathematical model for predicting the loss of renal allotransplant function can be realized in the tabular processor Excel and thus be used in medical practice.
5. For patients with delayed renal allotransplant dysfunction it is reasonable to restart the hemodialysis program at achieving plasma concentrations of creatinine of 0.5–0.7 mmol/L. At the same time hemodialysis immunosuppressive therapy must be canceled.

## Figures and Tables

**Table 1:** Comparative description of life quality indices of patients treated with hemodialysis and the recipients with a satisfactory function of kidney transplant.

Scales	Control (*n* = 50)	Dialysis (*n* = 62)	Transplants (*n* = 63)
Physical functioning	95.3 ± 9.7	67.4 ± 3.4	80.2 ± 5.8^*∗*^
Role, physical functioning	89.4 ± 8.7	45.4 ± 6.3	69.4 ± 8.8^*∗*^
Bodily pain	85.2 ± 5.4	65.5 ± 2.5	71.3 ± 5.3^*∗*^
General health	73.2 ± 6.2	43.5 ± 4.7	60.5 ± 6.1^*∗*^
Vitality	59.7 ± 4.9	49.1 ± 4.5	56.2 ± 4.6^*∗*^
Social functioning	85.0 ± 8.8	29.0 ± 3.2	45.3 ± 5.7^*∗*^
Role, emotional	63.1 ± 4.9	56.5 ± 2.5	58.7 ± 4.3^*∗*^
Mental health	62.8 ± 4.5	59.5 ± 3.5	60.7 ± 5.4

*Note*. *∗* means differences between groups of dialysis and transplantation patients are statistically significant (*P* < 0.05).

**Table 2:** Predictive value of basic clinical laboratory and sociodemographic variables of the survey one year after transplantation.

Factor sign	The value of the prediction *β* ± *m*	The level of significance differences from 0
Gender	0.021 ± 0.027	0.442
Age	0.024 ± 0.034	0.480
Cause of ESRD	0.017 ± 0.028	0.547
Donors: cadaveric/living	0.011 ± 0.033	0.740
Dialysis before transplantation	0.047 ± 0.03	0.125
Induction immunosuppression with anti-CD-25 monoclonal or other depleting antibodies	−0.035 ± 0.029	0.230
Hepatitis	−0.063 ± 0.029	0.030^*∗*^
Educational status	0.047 ± 0.035	0.183
Residence: town/village	0.002 ± 0.033	0.946
Number of transplantations	−0.017 ± 0.03	0.561
Creatinine	−0.277 ± 0.047	<0.001^*∗*^
Systolic blood pressure	−0.124 ± 0.055	0.024^*∗*^
Diastolic blood pressure	0.036 ± 0.045	0.436
Hemoglobin	0.265 ± 0.044	<0.001^*∗*^
Proteinuria	−0.394 ± 0.047	<0.001^*∗*^

*Note*. *∗* means more significant forecasting factor sign.
